# Manufacturing of Open-Cell Aluminium Foams: Comparing the Sponge Replication Technique and Its Combination with the Freezing Method

**DOI:** 10.3390/ma15062147

**Published:** 2022-03-15

**Authors:** Alina Sutygina, Ulf Betke, Michael Scheffler

**Affiliations:** Department of Mechanical Engineering, Institute for Materials and Joining Technology, Otto-von-Guericke-University Magdeburg, Große Steinernetischstraße 6, 39104 Magdeburg, Germany; ulf.betke@ovgu.de (U.B.); m.scheffler@ovgu.de (M.S.)

**Keywords:** aluminium foam, sponge replication, freezing, lamellar pores, specific surface area, thermal processing

## Abstract

The manufacturing of aluminium foams with a total porosity of 87% using the sponge replication method and a combination of the sponge replication and freezing technique is presented. Foams with different cell counts were prepared from polyurethane (PU) templates with a pore count per inch (ppi) of 10, 20 and 30; consolidation of the foams was performed in an argon atmosphere at 650 °C. The additional freezing steps resulted in lamellar pores in the foam struts. The formation of lamellar pores increased the specific surface area by a factor of 1.9 compared to foams prepared by the sponge replication method without freezing steps. The formation of additional lamellar pores improved the mechanical properties but reduced the thermal conductivity of the foams. Varying the pore cell sizes of the PU template showed that—compared to foams with dense struts—the highest increase (~7 times) in the specific surface area was observed in foams made from 10 ppi PU templates. The effect of the cell size on the mechanical and thermal properties of aluminium foams was also investigated.

## 1. Introduction

Open-cell metal foams are widely used in filters, heat pumps, as support for catalysis, as heat exchangers, etc. due to their thermal/electrical conductivity combined with high specific surface area and permeability for liquids and gases [1,2,3,4]. Depending on the application, these foams can be coated with active materials such as metal-organic frameworks (MOFs) or zeolites. Therefore, cell pore size, total porosity and specific surface area have a significant influence on the loading of active materials and the interaction efficiency of gases and liquids with the metal foam-based system, as well as the mechanical and thermal properties.

One of the processes for the manufacture of highly porous metallic foams with open-cell pores is the sponge replication method [5]. Metal foams produced by this method are characterized by a high total porosity. The porosity of such foams can easily be controlled by the cell size of a polyurethane (PU) template and the amount of metal powder in the suspension covering the PU template, which makes it possible to produce foams with a high specific surface area. Sponge replicated foams, in contrast to the more common methods of manufacturing metal foams by casting around hollow spheres, metal injection moulding, etc., are characterized by hollow struts formed after PU burn-out. The hollow struts also increase the specific surface area. However, this additional (inner) surface is difficult to cover with active materials and to contact with fluids and gases.

This obstacle may be solved by the formation of additional pores in the foam struts, which would give access to the hollow struts and additionally increase the specific surface area. Freeze-drying or casting is one of the methods of manufacturing porous structures [6]. By this method, ice crystals and areas of high concentration of solid particles are formed by freezing a suspension consisting of solid particles and a solvent (water, camphor or *tert*-butyl alcohol [6]). Sublimation of the frozen solvent produces a porous structure. Initially, sublimation freezing was used to make highly porous ceramics, but recently this method has also been applied to metal suspensions such as titanium [7,8], Ti-Al alloy [9], iron [10,11,12], copper [13,14,15,16], nickel foams [17,18,19] and Cu-Ni [17].

The feasibility of the combination of replication and freezing methods was first applied in [20] for aqueous SiO_2_ suspensions. The subsequent work of Schelm et al. in [21,22] for alumina foams showed a more detailed study of the effects of process parameters such as freezing temperature, solid loading, type of thickening agent on porosity, mechanical properties of samples and shape and size of pores. It was found that this combination of methods increased the total specific surface area of the foams from 70 to 200 cm^2^ g^−1^ due to the additional formation of lamellar pores. In [23], the production of metal foams based on copper powder made by this combination of methods was presented for the first time, which also showed the positive effect of the additional lamellar strut pores on the specific surface area and the mechanical properties of the foams. Despite the high density of copper powder and its rapid settling in suspension, the copper foams had lamella pores along with the entire sample.

Aluminium foams have unique properties such as high thermal and electrical conductivity, ductility and relatively low density compared to a large number of other metals, corrosion resistance and a relatively low melting point (~660 °C), which is an advantage when sintering foams from powders. Aluminium foams have been successfully produced by the sponge replication method [24,25], but despite quite promising results, a combination of sponge replication and freezing techniques has not been demonstrated for the production of aluminium foams in order to get accessibility to the hollow struts.

The aim of this work was to demonstrate the manufacturing of open-cell aluminium foams by a combination of sponge replication and freezing techniques. The additional freezing steps should result in additional strut porosity and should give access to the hollow inner cavities of the struts. This work is also aimed at investigating the effect of the cell size of a PU template and lamellar pores on the microstructure, porosity, specific surface area, mechanical and thermal properties.

The results presented within this paper are a part of a thorough investigation of the metal foam preparation by a combination of the established replica technique and the freeze-drying process. This process route includes a variety of process parameters, for example the chosen material itself (here: aluminium), the characteristics of the metal powder, the freezing conditions, or parameters for thermal consolidation of the samples. The cell size of the foam templates is one of these parameters, the effect of which has not been addressed in previous publications.

## 2. Materials and Methods

### 2.1. Specimen Preparation

As a starting material for the preparation of aluminium foams, an air-atomized aluminium powder, supplied by Ecka Granules (MEP103 RE903, Ecke Granules, Ranshofen, Austria), was used. This powder is characterized by a 99.5% purity with Ti < 0.25 wt.% and an average particle size of <10 μm.

**Figure 1:**
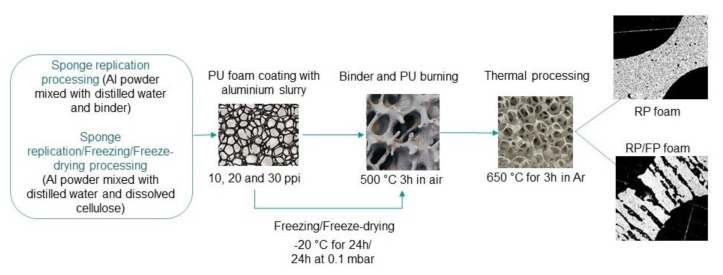
Schematic illustration of the samples preparation by sponge replication technique and a combination of sponge replication and freezing techniques; figure adapted from [23].

Figure 1 shows a schematic illustration of the samples preparation of replication processed foams (RP foams) and replication/freezed/freeze-dry processed foams (RP/FP foams). For the preparation of the RP foams, a 10.7 wt.% solution of a PVA binder (1.2 wt.%, Optapix PA 4G, Zschimmer and Schwarz Chemie GmbH, Lahnstein, Germany) in distilled water was taken. The aluminium powder was mixed with this solution in a planetary centrifugal mixer (THINKY Mixer ARE-250, THINKY Corp., Tokyo, Japan) at 2000 rpm for 6 min and cooled to room temperature. The resulting aluminium slurry contained 70.6 wt.% of aluminium. Open-cell PU templates (Koepp Schaum GmbH, Oestrich Winkel, Germany) were used to prepare RP aluminium foams. The PU templates were dipped into the metal slurry and manually squeezed to remove excess slurry from the PU foams. After that, the covered PU templates were dried for 24 h in air.

The next manufacturing step was the binder and PU template burn-out in air at 250 °C for 3 h and at 500 °C for 3 h in a circulating air furnace (KU 40/04/A, THERMCONCEPT Dr. Fischer GmbH, Bremen, Germany) with a heating rate of 1 K min^−1^. After that, the samples were thermally processed for 3 h in Ar atmosphere (purity 99.999%) at 650 °C in a conventional tube furnace (alumina tube, HTRH 70-600/1800, Carbolite-Gero GmbH and Co. KG, Neuhausen, Germany) with heating and cooling rates of 3 K min^−1^. The flow rate of Ar was 25 mL min^−1^.

For the preparation of the RP/FP foams, almost the same manufacturing route as for the RP foams was used but with an additional freezing and a freeze-drying step to obtain extra pores in the foam struts. The aluminium slurry for the RP/FP samples contained ~54 wt.% of solids and was prepared by mixing aluminium powder and distilled water with an additional thickener: methylcellulose (Tylose 3000 P, methyl hydroxyethylcellulose, Carl Roth GmbH & Co, KG, Karlsruhe, Germany) was dissolved in distilled water in a proportion of 1.5 g methylcellulose in 25 mL H_2_O. The aluminium powder was dispersed in this methylcellulose solution as described above. After coating the PU templates with the slurry and removing their excess, the samples were placed in a household freezer for 24 h to obtain water dendrites at −20 °C. Then, a freeze dryer (Gefriertrockenschrank BETA 1-16 LMC 2, Martin Christ Gefriertrocknungsanlagen GmbH, Osterode, Germany) was used to dry the RP/FP foams by water ice sublimation. This step was carried out for 24 h in a vacuum at 0.1 mbar at −50 °C to remove residual water in the as-formed lamellar strut pores. The further sample preparation of the RP/FP foams was the same as for the manufacturing of the RP foams, see Figure 1.

For the preparation of the RP and RP/FP samples, the PU templates with a different linear cell count of 10 ppi, 20 ppi and 30 ppi and a geometric size of 15 mm × 15 mm × 20 mm were used. For the measurement of the thermal conductivity, samples were manufactured from the PU templates with the geometric size of 20 mm × 50 mm × 50 mm.

### 2.2. Characterisation

A scanning electron microscope (SEM; FEI ESEM XL30 FEG, Hillsboro, OR, USA) was used to characterize the cross-sectional microstructure of the thermally processed samples. Before the cross-sectional analysis, samples were vacuum impregnated with epoxy resin, ground (180, 320, 600, 800, 800, 1200, 2500, 4000 mesh sandpaper) and polished (diamond suspension 3 µm and 1 μm).

The calculation of the total foam porosity (*P_total_*) was related on the geometrical foam density and the density of the foam strut material (*ρ_Al_* · 2.73 g·cm^−3^) using Equation (1):

(1) $$P_{total}=\frac{V_{pores}}{V_{foam}}=1-\frac{\;\rho_{geometr.}}{\rho_{Al}}$$

Archimedes’ method was applied to measure the strut porosity (*P_s_*) and the cell porosity (*P_cell_*). Water was taken as an infiltration fluid. The procedure was performed in accordance with DIN EN 623-2:1993-11 [26]. Prior to the measurement, aluminium foams were treated in an ultrasonic bath to fill the foam pores with water. A small amount of surfactant was added to reduce the surface tension. The weight of the dry, floating and wet foams was then measured.

An X-ray micro-computer tomograph (µ-CT) (Nanotom S, GE Sensing and Inspection Technologies, Wunstorf, Germany) was used to investigate the macrostructural features and to determine the geometric properties of the foams. Measurements were taken at an exposure time of 1250 ms per projection image. During the measurement of the samples, the voltage was 50 kV, and the tube current was 110 µA. The distance between the sample holder and the X-ray tube and detector was varied depending on the desired voxel size. For a complete foam measurement, the voxel size was 7 µm, for a small fragment of foam this value was up to 3.5 µm to obtain a higher resolution image. Data reconstruction was performed using the Phoenix Datos |X 2.0 software package (GE Sensing and Inspection Technologies, Wunstorf, Germany).

CT Analyzer 1.18 software (Skyscan/Bruker microCT, Kontich, Belgium) was used to determine the lamellar pore thickness, material lamellae thickness, and the surface areas were calculated from foam fragments that were determined from the µ-CT reconstruction data. The cell size distribution and the strut thickness distribution were calculated from measurements of complete foams. The average values for the size of lamellar pores, material lamellae and strut thickness (*D_s_*) were obtained from histograms by fitting Gaussian functions. More details about the calculation procedure can be found in [21,27]. In order to calculate the specific surface area per mass unit, aluminium tablets were produced similar to aluminium foam to determine the density of sintered aluminium. In this specific case, this density was ~1.63 g·cm^−3^. A more detailed calculation of the specific surface area is available in ref. [23].

For X-ray diffraction analysis (XRD), a D8 Discover diffractometer (Bruker-AXS GmbH, Karlsruhe, Germany, Co Kα 1/2 radiation) with Bragg–Brentano geometry and an energy-dispersive LynxEye-XE-T detector was used. The metal foam sample was compacted to a thin sheet using a hydraulic press and then placed into a backloading sample holder. The diffracted intensity was recorded in a 2θ range between 40° and 85° with a step size of 0.013° using a fixed divergence slit of 0.6 mm and a 2.5° primary and secondary soller slit. Phase analysis was performed with the Rietveld technique using the Topas V6 software package [28].

A TIRAtest 2825 uniaxial compression testing machine (TIRA GmbH, Schalkau, Germany) was used to investigate the mechanical properties (compressive yield strength and absorption energy). During the measurements, the samples were placed between two load plates with a diameter of 150 mm. The loading rate was 2 mm·min^−1^. The results of the average value of the compressive yield strength and the absorption energy were calculated from measurements of at least 10 samples using the Visual-XSel14.0 software package (CRGRAPH, Starnberg, Germany) [29]. The absorbed energy per unit volume *W* (MJ m^−3^) was calculated from Equation (2) and represents the integral area under the stress–strain curve [30]:

(2) $$W=\int_{0}^{\varepsilon_{D}}\sigma \left(\varepsilon \right)d\varepsilon$$

where *ε_D_* is the densification strain.

The Gibson and Ashby model (Equation (3)) given in ref. [30,31] was used to compare the relative compressive yield strength as a function of the foam density:

(3) $$\frac{\sigma }{\sigma_{b}^{*}}=C\cdot {\left(\frac{\rho^{*}}{\rho }\right)}^{n}$$

where *σ*—compressive yield strength, ρ*—foam density, *C*—proportionality constant, *n*—density exponent, σ_b_* and *ρ* are the yield strength and the density of bulk material, which were 29.4 MPa and 2.73 g·cm^−3^ for aluminium foams, respectively [32]. The proportionality constant *C* is related to the effect of the pore geometry. The density exponent *n* shows the mechanism of deformation.

The thermal conductivity of the foam (*λ_f_*) was analysed with the Hot Disk Transient Plane Source method (TPS 2500 S, Hotdisk SE, Gothenburg, Sweden). The size of the sensor was 9.868 mm in diameter. The sensor was placed between two ground foams to obtain a more uniform contact between the surfaces of the foam and the sensor. The heating power and heating time were varied according to the density of the foams and were set to 200 mW and 5 s. During the measurement process, the samples were turned over so that all combinations of sides were measured. Each combination of sides was measured for five times. The result was 20 measurements per batch of samples. The sensor temperature change was used to calculate the thermal conductivity [33].

A model derived by Ashby, Equation (4) [31], was applied to calculate the effect of the cell porosity on the thermal conductivity of the foams:

(4) $$\lambda_{s}=\frac{\lambda_{f}-P_{cell}\cdot \lambda_{g}}{1/3\cdot \left(1-P_{cell}\right)}$$

where *λ_s_* is the thermal conductivity of the porous strut material, *λ_g_* is the thermal conductivity of the gas phase (in our case it is air with 0.0264 W·m^−1^K^−1^ [31]), *P_cell_* is the cell porosity.

A model developed by Eucken [34] was used to calculate the thermal conductivity of the non-porous bulk strut material *λ_b_* in order to compare it with the thermal conductivity of pure aluminium:

(5) $$\lambda_{s}=\lambda_{b}\frac{1+2P_{s}\cdot \left(1-\frac{\lambda_{g}}{\lambda_{b}}\right)/\left(2\frac{\lambda_{g}}{\lambda_{b}}+1\right)}{1-2P_{s}\cdot \left(1-\frac{\lambda_{g}}{\lambda_{b}}\right)/\left(2\frac{\lambda_{g}}{\lambda_{b}}+1\right)}$$

where *P_s_* is the strut porosity without hollow strut pores, *λ_s_* is the thermal conductivity of the porous strut material, calculated from Equation (4).

## 3. Results

### 3.1. Comparison of the RP and RP/FP Aluminium Foams

**Figure 2:**
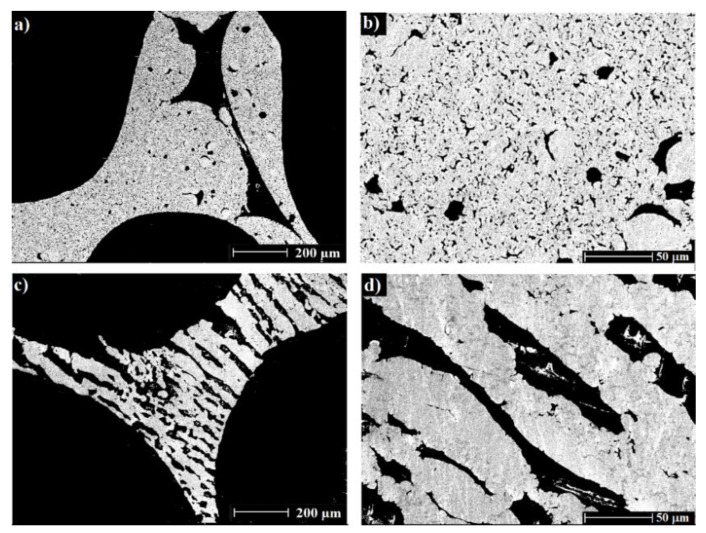
SEM images of the cross-sections of the RP (**a**,**b**) and RP/FP (**c**,**d**) foams after thermal processing.

Figure 2 shows SEM images of the cross-sections and foams surfaces of the RP and RP/FP aluminium foams. The RP foams are characterized by hollow struts formed after the PU burn-out and some pores inside the struts, as a result of thermal processing of aluminium powder (a,b). The foams prepared by a combination of sponge replication and freezing techniques showed the formation of lamellar pores and material lamellae, as this is typical for aqueous suspensions after freezing and freeze-drying, Figure 2c,d. These pores were randomly oriented because of non-directional freezing. In refs. [21,22,23], ceramic and copper foams manufactured by this combination were also characterized by a similar lamellar pore orientation behaviour.

The porosity in foams has a significant influence on the mechanical and thermal properties of the foams. Therefore, in this work foams with approximately the same porosity were prepared, in order to be comparable. Table 1 shows the results of porosity measurements by the Archimedes method. The total porosity (*P_total_*) of the foams was 86.6%. For the comparison of the RP and RP/FP foams, a PU template with a pore size of 20 ppi was used. Additional lamellar pores formed inside the struts increased the volumetric shrinkage from 41.9 ± 2.5% to 56.1 ± 3.7%, which decreased the cell porosity (*P_cell_*) from 80.9% to 73.5%. The RP/FP foams were characterized by a ~1.4 times higher strut porosity (*P_s_* ≈ 53.8%) compared to the RP foams (*P_s_* ≈ 37.6%). The strut porosity is the ratio of the volume of the material pores (*V_material pores_*) and the volume of the hollow struts (*V_hollow strut pores_*) to the volume of the struts consisting of the strut material (*V_material_*), the material pores and the hollow strut pores. The strut porosity was measured or calculated for struts only without the cell pores volume (*V_cell pores_*). That is why it is not reasonable to summarize cell porosity and strut porosity; this will result in a total porosity higher than 100%, cf. Table 1 and Table 2.

**Figure 3:**
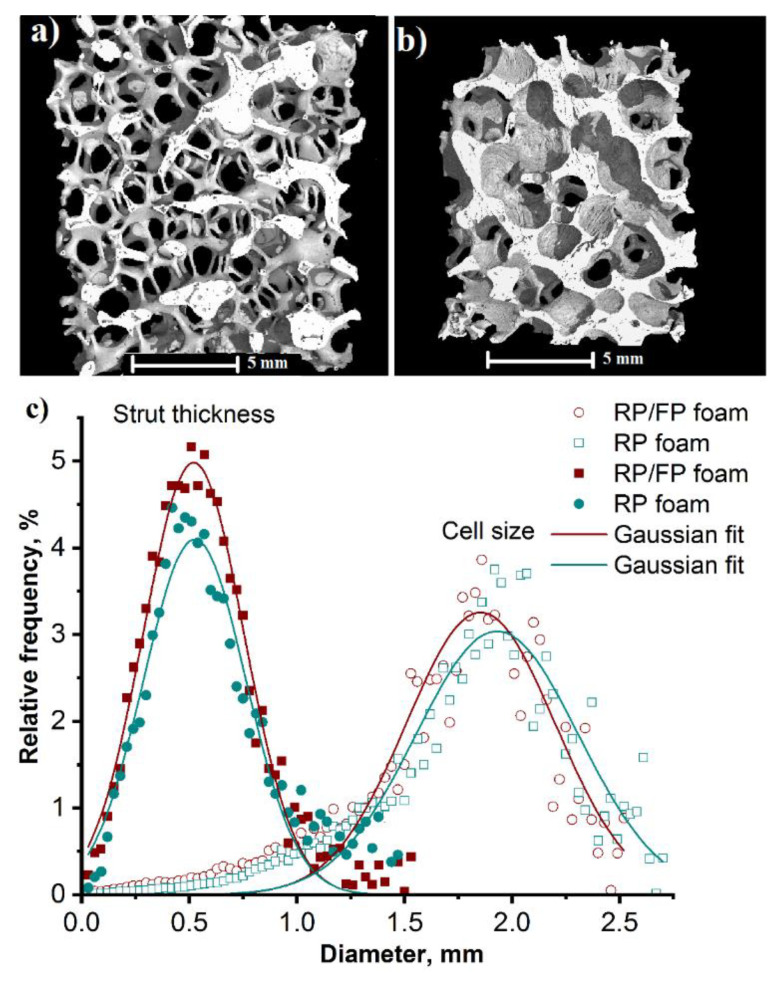
X-ray μ-CT 3D reconstruction image of the RP (**a**) and RP/FP (**b**) aluminium foams; distribution of cell size and strut thickness of the RP (green) and RP/FP (red) aluminium foams (**c**).

**Figure 4:**
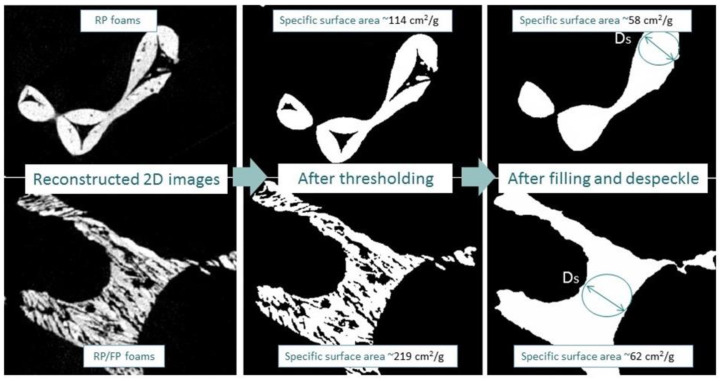
Illustration of reconstructed 2D images (**left**), after thresholding (**middle**) and filling and despeckle (**right**) steps of the RP (**top**) and RP/FP (**bottom**) foams.

Figure 3a,b shows 3D reconstructions of foams, which are characterized by open cells. The µ-CT measurements of the cell size (Figure 3c) showed that the distributions for the RP and the RP/FP foams were not significantly different. This was also evident from the mean cell size (1.96 ± 0.28 mm for the RP foams and 1.91 ± 0.22 mm for the RP/FP foams), determined by applying a Gaussian approximation to the distance histogram obtained from CTAnalyzer calculations of the CT data. The mean diameter of the struts was 0.48 ± 0.25 mm for the RP foams and 0.52 ± 0.23 mm for the RP/FP foams, Figure 3c. In Figure 4, the schematic illustrations of struts diameter (*D_s_*), calculated after thresholding and filling and despeckle procedures, are shown.

Figure 4 shows reconstructed 2D images of samples with micropores in the struts before (left) and after (middle) thresholding without pores in the struts and struts without hollow strut pores after filling and despeckle (right). These procedures were applied to estimate the influence of hollow strut and lamellar pores on the specific surface area. The calculations showed that the specific surface area increased significantly from 114 cm^2^·g^−1^ to 219 cm^2^·g^−1^ (~1.9 times) due to the additional lamellar pores formed after freezing. The specific surface area without hollow struts and lamellar pores (after filling and despeckle) for both samples did not differ significantly and was in the range of 58 cm^2^·g^−1^ (RP foams) to 62 cm^2^·g^−1^ (RP/FP foams). From these results it follows that the hollow struts increased the specific surface area for the RP foams by ~2 times, while for the RP/FP foams the specific surface area increased by ~3.5 times due to the additional lamellar pores and hollow strut pores.

**Figure 5:**
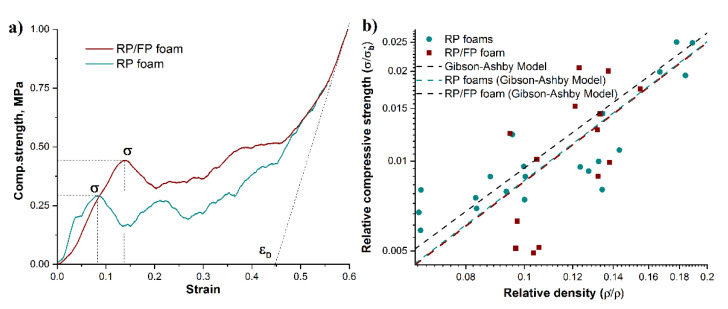
Compressive stress–strain curves of the RP and RP/FP aluminium foams (**a**); dependence of relative compressive strength and relative density of the RP (green dots) and RP/FP (red dots) foams; Gibson-Ashby models obtained by fitting experimental results with *C* and *n* variables (RP foams—green dash line and RP/FP foams—red dash line) and Gibson-Ashby model with fixed *C* = 0.3, *n* = 1.5 (black dash line) (**b**).

The mechanical properties of metal foams are significantly influenced by total porosity, pore size and pore type (open or closed), as well as the strut thickness [30,31,35]. Typical compressive stress–strain curves obtained for the RP and RP/FP foams are shown in Figure 5a, which demonstrate the elastic–plastic deformation behaviour typical for metal foams. The curves can generally be divided into an elastic deformation region (linear increase in the stress–strain curve), a plastic deformation region (plastic cells collapse as a result of yielding, buckling, or cell crushing) and a densification region (opposing cell walls start to come into contact and crush together) [30,36]. The compressive yield strength and the adsorbed energy were 0.34 ± 0.07 MPa and 0.10 MJ·m^−3^ for the RP foams and 0.49 ± 0.13 MPa and 0.15 MJ·m^−3^ for the RP/FP foams. The mechanical properties of the RP/FP foams were higher than for the RP foams, despite the existence of lamellar pores and a significantly higher strut porosity, which was expected to have a negative effect on the mechanical properties of the foams. This increase in mechanical strength might be connected with lamellar pore orientation. In ref. [37], it was observed that the direction and thickness of the lamellar pores affect the compressive strength, depending on their orientation with respect to the direction of load. The compressive strength generally increased with parallel lamellar pore direction to load but decreased with perpendicular pore orientation to load. In this work, the foams had a random lamellar pore orientation, which may have increased the strength characteristics, despite the high porosity of the strut, via a load sharing mechanism [38,39]. This behaviour of the mechanical properties has also been observed for copper foams [23].

Figure 5b shows a comparison of the relative compressive strength σ/σ_b_* as a function of the relative density ρ*/ρ_s_ for the RP and RP/FP foams; see also Equation (3). For metallic foams with open pores, the proportionality constant *C* and the density exponent *n* are usually close to 0.3 and 1.5, respectively [13,30,40,41]. However, these values can vary depending on the pore geometry, the deformation mechanism, etc. [39,42,43]. In order to compare the experimental values with the Gibson–Ashby model according to Equation (3), curves were plotted with a fix *C* = 0.3 and *n* = 1.5 (dashed line) and with a fix *C* = 0.3 and a varying fitted *n* value for the experimental results. For the RP and RP/FP foams, a Gibson–Ashby fitted curve with *n* ≈ 1.54 was obtained, which agrees well with the model. This discrepancy can be attributed to the fact that the Gibson–Ashby model was originally derived for open-cell foams with dense struts [30]. In this work, the aluminium foams were characterized by hollow struts as a result of the PU burn-out, as well as the lamellar pores formed during the freezing/freeze-drying step and pores in the strut material after incomplete thermal processing of the aluminium powder. It is known that porosity and pore size influence the mechanical behaviour of metal foams. It has been discussed that small pores can affect the mechanical strength via a load sharing mechanism [38,39]. This means that the compressive load can be distributed not only between each foam strut, but also between the individual material lamellae with a complex randomly oriented connection between them, which can consequently increase the withstand load of the foam, due to the impact of each material lamellae on it. All of the above may change the deformation modes of foams such as yielding, bending and buckling [30,44], and lead to a change in *n*. Further systematic studies of these foams are required to identify the deformation mechanisms.

**Figure 6:**
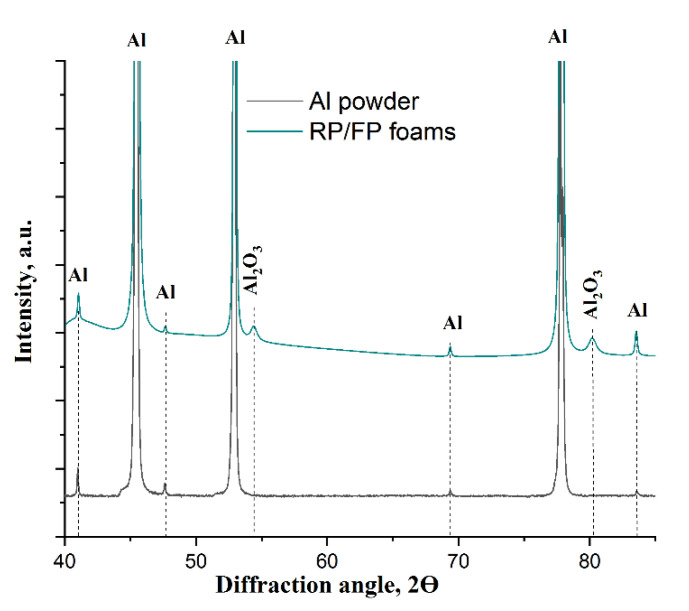
XRD patterns of the as-received powder (black) and the as-processed RP/FP foams.

The XRD phase analysis indicated the presence of the γ-Al_2_O_3_ phase obtained from aluminium foams after thermal processing (Figure 6). The amount of aluminium oxide was ~2.5 wt.% in the RP foams and ~3.0 wt.% in the RP/FP foams. The phase composition was determined using the Rietveld method. The aluminium foams were compressed before measurement to obtain a planar surface, from which the analysis was carried out.

The results of the measurement of the thermal conductivity coefficient *λ_f_* showed that the formation of additional lamellar pores reduced the thermal conductivity of the foams from 3.21 ± 0.18 W·m^−1^K^−1^ to 2.32 ± 0.05 W·m^−1^K^−1^. To estimate the effect of the strut porosity on *λ_f_*, the thermal conductivity coefficient of the porous strut material *λ_s_* was calculated according to Equation (4), which showed that *λ_s_* was ~61.7 W·m^−1^K^−1^ for the RP foams and ~32.1 W·m^−1^K^−1^ for the RP/FP foams. As a result, the lamellar pores reduced the thermal conductivity by a factor of ~1.9. The calculation of thermal conductivity of the bulk material *λ_b_* showed that the calculated values were ~98.4 W·m^−1^K^−1^ for the RP foams and ~80.3 W·m^−1^K^−1^ for the RP/FP foams. They were significantly lower than the thermal conductivity of pure bulk aluminium with ~205 W·m^−1^K^−1^ [45]. This decrease in *λ_b_* compared to pure aluminium bulk material was assigned to the presence of impurities in the thermally processed aluminium foams. In this work, these impurities are mainly caused by aluminium oxides in form of oxide shells around the powder particles that were also described in ref. [25]. Aluminium oxides are characterized by a low thermal conductivity of 24–39 W·m^−1^K^−1^ [46]. Another reason for the decrease in the thermal conductivity may be a change in the number of grain boundaries in the heat flow path. In metals with decreasing grain size, the thermal conductivity is lower as a result of the average free path of phonons decreasing, due to an increase in phonon scattering with decreasing grain size [47]. Further studies of these foams are required to study grain size effects on *λ_b_*.

### 3.2. Manufacturing of the RP/FP Aluminium Foams with Different Cell Size

Table 2 shows the results of shrinkage and porosity of the RP/FP aluminium foams processed with a PU template cell size of 10 ppi, 20 ppi and 30 ppi. After thermal processing, the foams were characterized by a relatively high volumetric shrinkage of 56–58%. For a further comparison of sample characteristics, foams with approximately the same total porosity of 86.5–86.9% were prepared. As the cell size in the original PU template decreased from 10 ppi to 30 ppi, there was a decrease in cell porosity from 76.8% to 68.6% and an increase in strut porosity from 47.3% to 59.8%. This increase in *P_s_* might be due to the fact that the porosity, in this case, included not only lamellar pores, but also hollow strut pores, the part of which was higher for the 30 ppi than for the 10 ppi templated foams.

**Figure 7:**
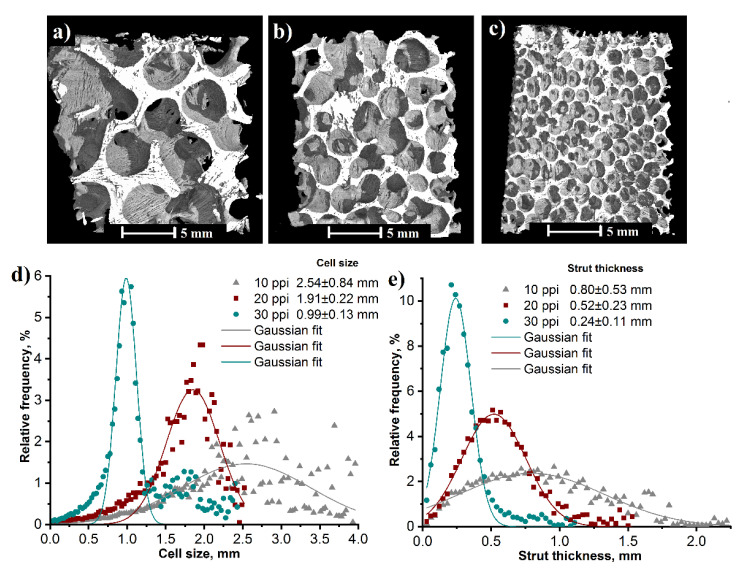
X-ray μ-CT reconstruction image of the RP/FP aluminium foams with the varied cell size of PU foams: 10 ppi (**a**), 20 ppi (**b**) and 30 ppi (**c**) and their distributions of cell size (**d**) and strut thickness (**e**) measured by μ-CT.

Figure 7 shows 3D µ-CT reconstruction images of the RP/FP aluminium foams with the varied cell size of the PU template and their distributions of cell size and strut thickness. From their calculated average values follows that the mean cell size and the mean strut thickness decreased with increasing value of ppi, therefore the distribution curves shifted to the left in Figure 7d,e. The cell size was 2.54 ± 0.84 mm for the 10 ppi, 1.91 ± 0.22 mm for the 20 ppi and 0.99 ± 0.13 mm for the 30 ppi templated foams. The strut thickness distributions (after thresholding, filling and despeckle) showed that *D_s_* varied from 0.24 ± 0.11 mm to 0.80 ± 0.53 mm with the increasing cell size of the PU template from 30 ppi to 10 ppi.

**Figure 8:**
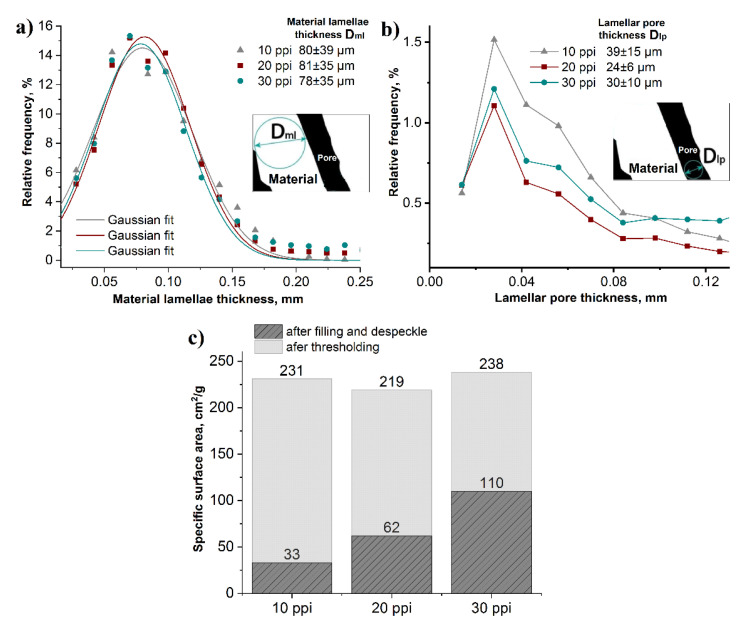
Size distributions of material lamellae (**a**), lamellar pores (**b**) and specific surface area (**c**) of the RP/FP aluminium foams templated with PU foams (10, 20 and 30 ppi); all results from µ-CT measurements.

The distributions of material lamellae, lamellar pores and specific surface area of the RP/FP foams in dependence on the cell size of the PU foams (10 ppi, 20 ppi and 30 ppi), measured by μ-CT is presented in Figure 8. The mean thickness of the material lamellae was 78–81 μm and the mean thickness of lamellar pores varied in size from 24 µm to 39 µm, Figure 8a,b. In order to estimate the influence of the hollow struts and lamellar pores on the specific surface area of the foams, the results of the calculations after thresholding and after the filling and despeckle were taken for comparison, Figure 8c. The additional lamellar pores and the hollow strut pores significantly increased the specific surface area, compared to foams with filled pores, from 33–110 cm^2^ g^−1^ to 219–238 cm^2^ g^−1^. The largest increase in specific surface area from 33 to 231 cm^2^ g^−1^ (~7 times) was observed for the sample made from the PU template with a cell size of 10 ppi. As the number of pores in the PU foam increased, the specific surface area after filling and despeckle increased also, due to the higher number of struts. Therefore, the specific surface area increased only by a factor of ~2.2 for the 30 ppi templated foams.

The compressive strength results in Table 3 showed that the compressive yield strength of the RP/FP aluminium foams with total porosity of 86.6–86.9% ranged from 0.44 ± 0.18 MPa (10 ppi) to 0.72 ± 0.19 MPa (30 ppi). The absorbed energy also increased with the number of pores in the PU template from 0.13 MJ·m^−3^ (10 ppi) to 0.29 MJ·m^−3^ (30 ppi), despite the increase in *P_s_* from 47.3% to 59.8%. This increase in the mechanical properties of the foams might be related to an increase in the number of struts in the samples, which improved the load distribution [14].

The results of the thermal conductivity study presented in Table 3 showed that *λ_f_* of the RP/FP aluminium foam was 2.18 ± 0.05 W·m^−1^K^−1^ for the 10 ppi, 2.32 ± 0.05 W·m^−1^K^−1^ for the 20 ppi and 2.97 ± 0.18 W·m^−1^K^−1^ for the 30 ppi templated foams. The calculation of the thermal conductivity of the porous strut material showed that *λ_s_* did not change significantly when the pore size of the original PU template was changed, and it was 32.1–34.8 W·m^−1^K^−1^.

## 4. Conclusions

Aluminium foams with a total porosity of 86.5–86.9% were obtained by sponge replication and a combination of sponge replication and freezing techniques after consolidation at 650 °C in argon atmosphere. Foams with different cell sizes were prepared from PU templates with a pore count of 10 ppi to 30 ppi. The freeze processed foams possess lamellar pores with a thickness of 24–39 µm distributed along with the entire sample, but with random orientation. The formation of additional lamellar pores increased the strut porosity by ~1.4 times and the specific surface area increased by ~1.9-times compared to foams prepared by the conventional sponge replication method. Varying the pore size of the initial PU foams from 10 ppi to 30 ppi showed that the specific surface area of the foams obtained by a combination of sponge replication and freezing techniques slightly changed with the cell size from 219 to 238 cm^2^ g^−1^.

The formation of additional porosity increased the absorbed energy and the compressive yield strength, despite the significant increase in strut porosity. The relative compressive strength as a function of relative density agreed well with the Gibson–Ashby model for the foams prepared by both techniques. With the increasing number of cell pores in the initial PU template, an increase in the mechanical properties was observed.

The increase in strut porosity after the formation of lamellar pores negatively affected the thermal conductivity of the foams. The thermal conductivity coefficient was higher for the samples with smaller cells.

## Figures and Tables

**Table 1:** Porosity of the RP and RP/FP aluminium foams with a PU template cell size of 20 ppi.

Sample	Total Porosity ^a^, %	Cell Porosity ^b^, %	Strut Porosity ^c^, %
RP foam	86.6 ± 0.7	80.9 ± 1.5	37.6 ± 2.7
RP/FP foam	86.6 ± 1.1	73.5 ± 2.5	53.8 ± 3.4

^a^ (*V_material pores_* + *V_hollow strut pores_* + *V_cell pores_*)/*V_foam_*; ^b^ (*V_cell pores_* + *V_hollow strut pores_*)/*V_foam_*; ^c^ (*V_material pores_* + *V_hollow strut pores_*)/(*V_hollow strut pores_* + *V_material_* + *V_material pores_*).

**Table 2:** Shrinkage and porosity of the RP/FP aluminium foams with a PU template cell size of 10 ppi, 20 ppi and 30 ppi.

Sample	Volumetric Shrinkage, %	Total Porosity ^a^, %	Cell Porosity ^b^, %	Strut Porosity ^c^, %
10 ppi	57.6 ± 2.8	86.9 ± 0.9	76.8 ± 2.1	47.3 ± 2.7
20 ppi	56.1 ± 3.7	86.6 ± 1.1	73.5 ± 2.5	53.8 ± 3.4
30 ppi	56.4 ± 1.4	86.5 ± 0.6	68.6 ± 4.1	59.8 ± 4.5

^a^ (*V_material pores_* + *V_hollow strut pores_* + *V_cell pores_*)/*V_foam_*; ^b^ (*V_cell pores_* + *V_hollow strut pores_*)/*V_foam_*; ^c^ (*V_material pores_* + *V_hollow strut pores_*)/(*V_hollow strut pores_* + *V_material_* + *V_material pores_*).

**Table 3:** Mechanical and thermal properties of the RP/FP aluminium foams with a PU template cell size of 10 ppi, 20 ppi and 30 ppi.

Sample	Comp. Yield Strength, MPa	Absorbed Energy, MJ·m^−3^	*λ_f_*, W·m^−1^K^−1^	*λ_s_*, W·m^−1^K^−1^
10 ppi	0.44 ± 0.18	0.13	2.18 ± 0.05	34.6
20 ppi	0.49 ± 0.13	0.15	2.32 ± 0.05	32.1
30 ppi	0.72 ± 0.19	0.29	2.97 ± 0.18	34.8

## Data Availability

All relevant data are mentioned in the publication.
